# Sustainable Nutrition and Human Health as Part of Sustainable Development

**DOI:** 10.3390/nu16020225

**Published:** 2024-01-10

**Authors:** Magdalena Gibas-Dorna, Wioletta Żukiewicz-Sobczak

**Affiliations:** 1 Collegium Medicum, Institute of Health Sciences, University of Zielona Gora, 28 Zyty Street, 65-046 Zielona Gora, Poland; 2 Department of Nutrition and Food, Faculty of Medical and Health Sciences, Calisia University, 4 Nowy Swiat Street, 62-800 Kalisz, Poland; 3 Department of Biological Bases of Food and Feed Technologies, University of Life Science in Lublin, 28 Głęboka Street, 20-612 Lublin, Poland

The concept of sustainable nutrition is focused on an optimal and health-promoting diet that is culturally acceptable, easily accessible, and eco-friendly by reducing environmental costs for present and future generations. In 1986, Gussow and Clancy, for the first time, indicated that nutrition education and individual nutritional choices should not be based only on medical knowledge. Environmental, macroeconomic, and agricultural factors must be considered to protect natural food resources and care about the health of ecosystems [1]. To support life and health, the WHO and FAO organizations released the document entitled “Sustainable healthy diets–Guiding principles”, which implements the role of a healthy diet in creating environmentally sustainable food systems [2]. According to this document, the guiding principles refer to the following three main issues: (1) health aspects, which highlight the role of breastfeeding in early life, the use of unprocessed or minimally processed foods free of agents that can cause foodborne and non-communicable diseases (NCDs), (2) environmental impacts by reducing and maintaining set targets for gas and chemical pollution, water and land use, and the preservation of the biodiversity of food products, (3) sociocultural aspects, making food accessible and affordable to all members of the population, considering local culture and culinary practices. To meet the complexity of these aspects, building the knowledge base of multidisciplinary determinants and potential interventions is necessary. Research on adopting environmentally sustainable and healthy diets is in progress, but to achieve consensus for global policymaking, interventions increasing the convergence of science, released recommendations, economics, and consumer-oriented needs must be put into practice. For example, in September 2015, the United Nations adopted the 2030 Agenda with the Sustainable Development Goals [3], which has been used to develop the EU’s investment framework for external action with specified targets and methods of its successful implementation [4].

Understanding the importance of sustainable nutrition and its possible influence on well-balanced and healthy population development, we launched this research topic named “Sustainable Nutrition and Human Health as Part of Sustainable Development”. After 17 months of accumulating contributions, 11 full papers exploring the link between sustainable nutrition and sustainable development were accepted, including 8 research articles and 3 reviews. These papers concern global trends in nutritional deficiencies among children, the external costs of nutrition, consumers’ awareness of environmental and dietary sustainability, eating behaviors, including the COVID-19 pandemic period, and possibilities to reuse waste products. 

According to the goals of a sustainable diet, in some low-income countries, global nutritional deficiencies and undernutrition are major public health concerns. Based on the Global Burden of Disease Study database, Yue et al. identified changing trends of nutritional deficiencies among children under 5 years of age from 2010 to 2019 (contribution 1). The authors analyzed the main subcategories of nutritional deficiencies, including the incidence of nutritional deficiency, disability-adjusted life years (DALYs), and the estimated annual percentage change (EAPC). Although both global incidence and DALY rates declined significantly, the burden of nutritional deficiency and persistent malnutrition in some African countries, particularly in Western and Central Sub-Saharan Africa, remains a challenge for health systems at the national and international levels.

To protect the environment and human health, raising public awareness can play an important role in consumer engagement. As indicated by Rossi et al., dietary strategy consensus and food policies should contain practical guidance on sustainability based on cooperation between public health nutrition and ecology experts (contribution 2). In the study performed by Chang et al., the authors investigated consumers’ awareness of environmental and dietary sustainability (contribution 3). They found that health-conscious Taiwanese consumers purchasing healthy boxed meals were interested in buying boxed meals labeled by traceability certification and nutrition facts, including the carbon footprint label of dietary products. In another study, Chang et al. described the need to label products with health-food labels to educate consumers about product attributes and increase healthy product intake (contribution 4).

Promoting organic food and reducing meat ingestion is one of the strategies that fits into environmentally sustainable food consumption. However, it is difficult to change nutritional habits, especially in a population influenced by a culture around eating meat, even if the knowledge about healthy lifestyle practices is indisputable. Based on the surveys conducted in urban and rural areas, Balan et al. explored Romanian consumers’ behavior and eating habits (contribution 5). The results revealed that the overconsumption of animal products, starchy vegetables, bread, and pastry products is in apparent conflict with the concepts of sustainable nutrition and development. More consumer-oriented interventions, including nutritional education, are required to reverse this situation. Chen H.-S. studied the factors influencing the intentions of young Taiwanese consumers to consume artificial meat products (plant-based meat). He described a significant relationship between consumers’ desire and several behavioral factors (e.g., subjective norms, behavioral intention, consumer-perceived behavioral control, positive anticipated emotions, and consumers’ environmental concerns) that could be modified to achieve the desirable effect (contribution 6). The economic costs due to unsustainable nutrition and negative health effects are important factors that may determine desirable changes in feeding behavior. Seidel et al. estimated the health costs of harmful nutrition associated with increased risks of diet-related NCDs. Almost one-third of these costs were due to the overconsumption of meat. Thus, the authors suggest that including the external costs of nutrition in actual food prices can decrease the demand for meat and other high-risk products and promote sustainable food consumption (contribution 7).

Two other articles in this Special Issue concern nutritional behaviors during and after the COVID-19 pandemic, which was a critical period that provoked a change in dietary patterns and public interest in food sustainability. The impact of the COVID-19 pandemic on the public’s interest in food and general sustainability in 13 European countries was investigated by Portugal-Nunes et al. Based on the relative search volume (RSV) of queries conducted using Google Trends, the authors observed that after the COVID-19 outbreak, public interest in environmental sustainability increased, but not as much as awareness of food sustainability (contribution 8). Interestingly, in many European countries and depending on the studied population, this positive attitude towards sustainable nutrition did not mirror real eating behaviors, which was also presented in a narrative review by Wiśniewski et al. (contribution 9).

Reducing and reusing waste when appropriate is an important target for sustainable nutrition and development. Upcycled food waste may provide nourishment to those in need and bring tangible economic benefits. Two papers focus on this issue. In a comprehensive review, Fărcaș et al. describe the beneficial properties of bioactive compounds derived from cereal by-products and their possible dietary application (contribution 10). Ummels et al. examine the usefulness of brewer’s spent grain (BSG) as a novel potential protein source suitable for human consumption. In a double-blind, cross-over intervention trial, they evaluate the postprandial amino acids uptake kinetics of barley/rice protein (BRP) in a healthy adult population, aiming to assess BRP protein quality. They found that BRP is characterized by good absorption with a high postprandial uptake of phenylalanine, methionine, and tryptophane (contribution 11). 

Unfortunately, processed food and inappropriate eating habits are becoming an everyday reality in our civilization, while diet-related diseases cause several important pathological processes in the human body. Unfortunately, the real problem is that lifestyle diseases create a vicious circle. [5,6]. The incidence of one disease increases the likelihood of developing another. In the 1970s, the Canadian Minister of Health LaLonde developed a concept containing four factors determining health (lifestyle accounted for 53%, physical and social environment approximately 21%, genetic predispositions approximately 16%, and the healthcare system only 10%). In turn, health behaviors are divided into health-promoting ones, which build health potential, and anti-health behaviors, which include, among others, smoking, a diet rich in fats and sugars, and stress [7]. For this reason, quick and substantive actions toward sustainable management and the existence of the population are necessary. The farm-to-plate strategy, or establishing a planetary diet by the European Green Deal strategy, is an opportunity to improve many important issues in human life and health [8]. Starting with waste-free agricultural production, through the use of well-balanced eating habits consistent with the rich knowledge we already have and which is periodically developing in many research areas, there is a chance to eliminate many serious problems in the population [9].

## References

[1] Gussow J.D., Clancy K.L. (1986). Dietary guidelines for sustainability. J. Nutr. Educ..

[2] FAO, WHO (2019). Sustainable Healthy Diets—Guiding Principles.

[3] https://sdgs.un.org/2030agenda.

[4] https://finance.ec.europa.eu/publications/sustainable-finance-package-2023_en.

[5] Frąszczak K., Barczyński B., Kondracka A. (2022). Does Lactobacillus Exert a Protective Effect on the Development of Cervical and Endometrial Cancer in Women?. Cancers.

[6] Barczyński B., Frąszczak K., Grywalska E., Kotarski J., Korona-Głowniak I. (2023). Vaginal and Cervical Microbiota Composition in Patients with Endometrial Cancer. Int. J. Mol. Sci..

[7] Lalonde M. (1974). A New Perspektive on the Heathl of Canadians; A Working Document.

[8] https://www.thelancet.com/commissions/global-syndemic.

[9] https://eur-lex.europa.eu/legal-content/PL/TXT/?uri=COM%3A2019%3A640%3AFIN.

